# Crucial factors preceding compulsory psychiatric admission: a qualitative patient-record study

**DOI:** 10.1186/s12888-017-1512-y

**Published:** 2017-10-24

**Authors:** Mark H. de Jong, Margreet Oorschot, Astrid M. Kamperman, Petra E. Brussaard, Esther M. Knijff, Roland van de Sande, Arthur R. Van Gool, Cornelis L. Mulder

**Affiliations:** 1 Yulius Academy, Yulius Mental Health, Dennenhout 1, 2994 GC Barendrecht, The Netherlands; 2 Epidemiological and Social Psychiatric Research Institute (ESPRi), Department of Psychiatry, Erasmus University Medical Centre, Postbus 2040, 3000 CA Rotterdam, The Netherlands; 3 Faculty of Health, University of Applied Science Utrecht, Bolognalaan 101, 3584 CJ Utrecht, The Netherlands

**Keywords:** Compulsory admission, Schizophrenia, Community mental health care, Qualitative research

## Abstract

**Background:**

Compulsory admissions have a strong effect on psychiatric patients and represent a deprivation of personal liberty. Although the rate of such admissions is tending to rise in several Western countries, there is little qualitative research on the mental health-care process preceding compulsory admission. The objective of the study was to identify crucial factors in the mental health-care process preceding compulsory admission of adult psychiatric patients.

**Methods:**

This retrospective, qualitative multiple-case study was based on the patient records of patients with severe mental illness, mainly schizophrenia and other psychotic disorders. Twenty two patient records were analyzed. Patients’ demographic and clinical characteristics were heterogeneous. All were treated by Flexible Assertive Community Treatment teams (FACT teams) at two mental health institutions in the greater Rotterdam area in the Netherlands and had a compulsory admission in a predefined inclusion period. The data were analyzed according to the Prevention and Recovery System for Monitoring and Analysis (PRISMA) method, assessing acts, events, conditions, and circumstances, failing protective barriers and protective recovery factors.

**Results:**

The most important patient factors in the process preceding compulsory admission were psychosis, aggression, lack of insight, care avoidance, and unauthorized reduction or cessation of medication. Neither were health-care professionals as assertive as they could be in managing early signs of relapse and care avoidance of these particular patients.

**Conclusion:**

The health-care process preceding compulsory admission is complex, being influenced by acts, events, conditions and circumstances, failing barriers, and protective factors. The most crucial factors are patients’ lack of insight and cessation of medication, and health-care professionals’ lack of assertiveness.

**Electronic supplementary material:**

The online version of this article (10.1186/s12888-017-1512-y) contains supplementary material, which is available to authorized users.

## Background

Compulsory admissions have a strong effect on psychiatric patients and their relatives, and can even be traumatic [1]. They are also contrary to human rights and to the principles of shared decision-making and recovery-focused care [2, 3]. During compulsory hospitalization, 30–50% of the patients undergo coercive interventions, such as enforced medication, seclusion and restraint [4]. Fearing coercion, some may therefore stay away from treatment [5]. A selection of very dissatisfied patients described coercive care in strong terms, such as humiliation, oppression and imprisonment by totalitarian systems [6]. In a prospective study, however, perceived coercion decreased significantly during hospitalization: at discharge, most patients (87%) reported that even though they had felt coerced during their stay, their admission had been justified [7]. Compulsory admission has also been associated with improvements in psychosocial functioning and better motivation for treatment [8]. Overall, compulsory psychiatric admission is commonly seen as unavoidable in patients whose psychiatric condition makes them a severe danger to themselves or to others [9].

In several European countries including the Netherlands, rates of compulsory admission are tending to rise [10–12]. Various factors may explain this rising trend, including shorter hospital stays (if patients are discharged from hospital earlier, they are presumably readmitted more often); increased provision of community mental health care (i.e. more individuals living in the community are identified as psychiatric patients); and urbanization [13]. Tolerance of behaviour that the general public labels as deviant (such as wearing dirty clothes or behaving strangely in the streets) seems to be decreasing, at least in Western countries. This might also lead rates of compulsory admission to rise [14].

A Dutch study among psychiatric crisis patients found the following factors to be associated with a higher risk of compulsory admission: previous compulsory admission, living alone, and patients’ dissatisfaction with the mental health care they received [15]. An Italian study among schizophrenia-spectrum patients found that previous compulsory admission, drop-out from mental health care, severity of illness, positive symptoms, excitement, emotion perception, and insight all differed significantly between patients who had been admitted compulsorily and those admitted voluntarily, This study stressed the importance of giving patients proper attention during the phase in which their emotional perception and insight are diminished [16].

While human rights considerations and the rising rates of compulsory admissions both indicate the urgent need to reduce compulsory admission rates, there has been little qualitative research on the mental health-care process that precedes compulsory admission. A study focussing on the family perspective showed that family members experience relief and conflicting emotions when a relative is admitted compulsorily [17]. Greater understanding of important factors in the health-care process that precedes compulsory admission might help to target interventions for reducing these admission rates. To our knowledge, however, the perspective of health-care professionals has not been used for detailed qualitative analysis of the process preceding compulsory admissions.

### Objective

In this study we therefore aimed to identify crucial factors in the health-care process preceding compulsory admissions of adult psychiatric patients. By “health-care process” we understand the whole process of service provision, including factors regarding patients and their relatives, health-care professionals, and the context of the local mental health care.

## Methods

### Qualitative approach

**Fig. 1:**
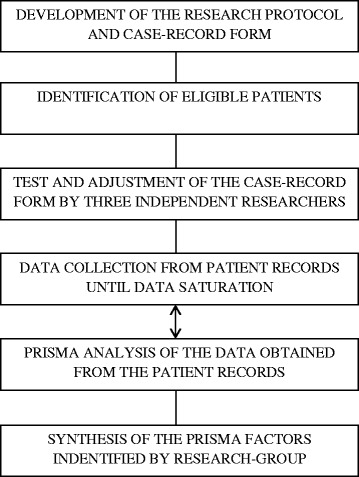
Flow chart of the qualitative research process

We based this retrospective multiple case study on detailed and structured analysis of patient records, assuming that this would increase our general understanding of the mental health-care process that precedes compulsory admission. This method reflects an interpretivist paradigm. We summarized all main steps in the research process in a flow chart (Fig. 1).

### Researcher characteristics

The following researchers were involved in the patient record analysis: a psychologist (MO), a psychiatry resident (PB), and two psychiatrists (EK, MJ). All had relevant clinical experience. To prevent biased analysis of the processes in question, no researchers studied the records of patients they themselves had treated.

### Context

The records studied were those of patients treated by the Flexible Assertive Community Treatment (FACT) teams [18] at Yulius and Bavo Europoort, two mental health institutions in the greater Rotterdam area, which cover various urban and outlying areas. The FACT teams provide assertive community treatment for unstable and care-avoiding patients; and coordinated, multidisciplinary treatment and recovery-oriented care for stable long-term patients. The mean number of patients per team is 200. They are treated for severe mental illness, mainly schizophrenia and other psychotic disorders. As patients with severe mental illness have the highest risk of being admitted compulsorily, they are logically the most important target group for interventions intended to reduce compulsory admission rates.

The teams consist of doctors, nurses, social workers, psychologists, and peer workers. All team members generate entries in the electronic patient records, which consist of reports on regular home visits and appointments at the office, crisis reports, notes of multidisciplinary meetings, psychiatric, psychological and physical assessments, treatment plans, treatment plan evaluations, crisis plans, medication prescriptions, judicial procedures, and correspondence. Basically, all activities with or regarding the patient are recorded in the patient record. Since the information and reports in the patient records are usually generated by the health-care professionals, the records reflect the professionals’ views and interpretations of the patient’s current situation.

### Sampling strategy

All FACT patients who had been compulsorily admitted to one of these institutions during the reference period were eligible. Using the lists of consecutive admissions derived from the institutions’ databases, we started with the latest compulsory admission (reference date 1 September 2014), and then tracked them in reverse order. Most compulsory admissions included in this study occurred within the 6 months prior to the reference date. Data collection was stopped after 22 patient records had been studied, as no more new information emerged from the records: in other words, data saturation had been reached [19]. Patient demographic and clinical characteristics are summarized in Table 1.

**Table 1:** Baseline demographics and clinical characteristics of all included patients (*N* = 22)

	Mean / N	Range / %
*Baseline demographics*		
Age	39.7	21–59
Sex		
Female	11	50
Male	11	50
Employment		
Yes	0	0
Marital status		
Living alone	14	64
Married	3	14
Divorced	1	4
Living with parent(s)	4	18
*Clinical characteristics*		
DSM-IV Axis I		
Schizophrenia	12	55
Psychotic disorder NAO	6	27
Schizoaffective disorder	2	9
Delusional disorder	1	4
Bipolar disorder	1	4
Duration of treatment in mental health care (years)	8.4	0–24
Previous compulsory admission	15	68
Compulsory admission procedure		
Emergency admission	17	77
Court-ordered admission	5	23
Most important danger		
To self	11	50
To others / environment	11	50
GAF^1^ at admission	32.7	25–45
Crisis plan present	3	14
Medication total	20	91
Antipsychotics (all)	19	86
Antipsychotics (depot)	2	9
Antidepressants	4	18
Mood stabilizers	1	4
Benzodiazepines	12	55
Other	8	36
Substance abuse total	12	55
Alcohol	6	27
Cannabis	8	36
Cocaine	3	14
Amphetamines	1	4

^1^ *GAF* Global Assessment of Functioning

### Ethical issues and data processing

Under the Netherlands’ Agreement on Medical Treatment Act (Dutch abbreviation WGBO), patient record research does not require patients’ informed consent if individual patients cannot be identified on the basis of the data. Our study was approved by an accredited Medical Research Ethics Committee (MREC), which classified it as outside the scope of the Netherlands’ Medical Research Involving Human Subjects Act (Dutch abbreviation WMO), and which confirmed that no informed consent was required. As required under the applicable Dutch laws, all researchers involved were bound to strict confidentiality.

According to our protocol, the patient record numbers were irreducibly coded to a research number by a research assistant at the Research Bureau (Yulius) who was not involved in the research itself and had no access to patient records. The coding list was stored at the Research Bureau and was inaccessible to all researchers involved. All data derived from patient records were carefully anonymized and saved according to the research numbers.

### Data collection methods

The data were collected on the basis of a detailed case-record form. Three researchers (EK, MJ, MO) used two patient records to test its first version for feasibility and agreement on its interpretation. Next, in a consensus meeting, they used these initial findings to adjust the case-record form, whose definitive version was used for analysis of all further patient records. Each of these researchers analysed different patient records, collecting socio-demographic and clinical data such as age, sex, employment and marital status, and also data on duration of treatment in mental health care, former compulsory admissions, diagnosis, medication, and substance abuse.

The Netherlands’ Exceptional Admissions to Psychiatric Hospitals Act (Dutch abbreviation BOPZ) has two main procedures for compulsory admission: emergency admission sanctioned by the mayor of the town in question and court-ordered admission. We therefore noted the type of compulsory admission.

The key step in the data collection process was as follows: starting 6 months prior to the admission date, we scrutinized all the reports in the patient records made by all the healthcare professionals involved, noting all information seen as relevant to compulsory admission: patients’ behaviour (e.g. symptoms and medication use), the activities of the health-care professionals in the FACT teams, all relevant events and occasions, and other potential contributory factors.

### Data analysis

All data retrieved in the initial process were then analysed according to the Prevention and Recovery System for Monitoring and Analysis (PRISMA) method, a method for critical incident analysis [20]. Such analysis starts with the “incident”, which in our study was compulsory admission. The core characteristic of the PRISMA method is to identify causes and factors underlying the incident. There are four distinctive types of factors: acts or events (red), conditions and circumstances (orange), failing protective barriers, i.e. factors that should provide some kind of protection, whereas in a given situation they did not (blue), and finally recovery or protective factors, i.e. factors that slowed the process preceding the incident (green). Each cause or factor may have another underlying cause or factor. In this way, a “tree of causes” was drawn for each of the included patients.

### Techniques to enhance reliability

As stated above, the case-record form was finalized after a consensus meeting between three researchers. The rough material obtained from all included patient records was independently assessed and summarized in the PRISMA tree of causes by two researchers (MJ, PB). Factors independently identified by these two researchers in the same case were deemed to be crucial to the health-care process that preceded that patient’s compulsory admission. By consensus, the supervising research group decided on data saturation and synthesized the commonest patterns and characteristics of the PRISMA factors we identified on the basis of all trees of causes.

## Results

### Crucial factors identified by critical incident analysis

In accordance with the PRISMA method, all the factors identified - independently by both researchers involved - in the health-care processes preceding the compulsory admissions (*N* = 22) were allocated to four categories. First, we identified acts and events. Aggression and unauthorized reduction or cessation of medication were relevant in most patients. Some patients showed self-neglect leading to serious medical conditions such as severe weight loss or infections. Further important factors were transfers to other health-care teams and life events such as the death of a close family member.

Second, we identified the patients’ conditions. Psychosis and care avoidance of mental health care characterized nearly all the patients, and was often accompanied by lack of insight into the psychiatric condition. Other relevant conditions in this category were paranoid thinking or delusions, substance abuse, medication side-effects, manic symptoms, suicidality, family history of psychiatric disorders, imperative hallucinations, polypharmacy, and intellectual disability.

Third, the failing protective barriers were as follows: health-care professionals did not respond assertively enough to signs or poor service engagement on the part of the patient, and did not supervise medication administration sufficiently. For example, in the event of missed appointments and other signs of care avoidance, professionals made phone calls rather than home visits. If a patient did not answer a phone call immediately, the professional tended to wait a day or more rather than travelling to the patient’s home and knocking on the door. Similarly, rather than proactively seeking contact, they merely responded to signals from involved family members. Other relevant factors in this category were slow or unclear judicial procedures, unclear diagnosis, insufficient reporting by health-care professionals in the patients’ records, and a limited social-support system.

Fourth, we identified protective factors that were used to reduce the risk of compulsory admission: the involvement of family members, inviting them to participate in the treatment process, voluntary admissions as a preventive strategy, strict supervision of medication administration by nurses and / or family members, sheltered housing, support with financial affairs and debt prevention, and increasing the frequency and duration of mental health-care contacts.

**Fig. 2:**
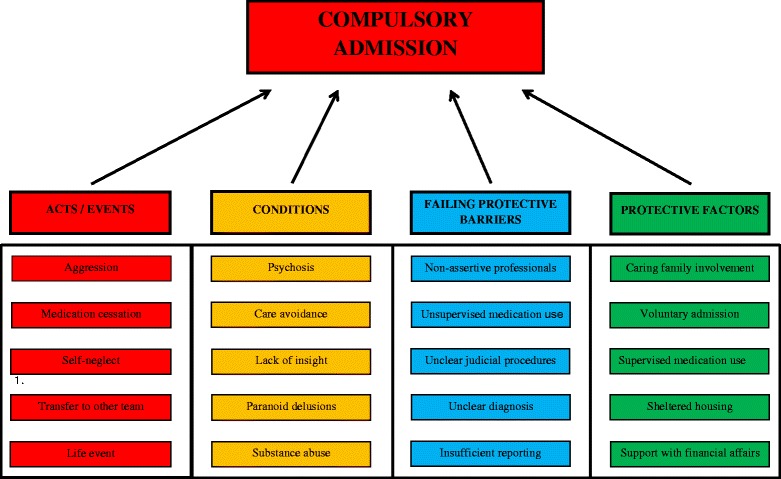
Crucial factors in four PRISMA categories

These crucial factors of the four PRISMA categories are summarized in Fig. 2. We also present an anonymized example of a tree of causes according to the PRISMA factors identified in the data (see Additional file 1). This should be read by starting at the top and asking “Why?” on each arrow pointing downwards towards the next factor.

### Additional findings

To identify patterns in subgroups, we divided patients on the basis of several characteristics. The distribution of PRISMA factors showed no differences with regard to the following patients: those with substance abuse versus those without, those with an emergency compulsory admission versus those with a court-ordered compulsory admission, those with a previous compulsory admission versus those without, those living alone versus those not living alone, those with a crisis plan and those without, and those using oral antipsychotics versus depot antipsychotics. Under the Dutch judicial system, compulsory admission is permissible only in people whose psychiatric condition endangers themselves or others. On the basis of eight predefined dangerousness criteria, psychiatrists examining patients at compulsory admission must therefore indicate applicable danger criteria on a default form, also indicating which is the most important. We found that patients who represented the greatest danger to others were more likely to show paranoid thinking (*N* = 6) and substance abuse (*N* = 4) than those who represented the greatest danger to themselves (*N* = 2 and *N* = 2, respectively). However, they were less likely to lack sufficient supervision of medication administration (*N* = 1 vs. *N* = 6).

### Anonymized case example

Patient A, a 47-year old female diagnosed with paranoid schizophrenia, became psychotic after unauthorized cessation of her antipsychotics. She became increasingly avoidant towards health-care professionals. They tried to contact her by phone for several days. Then, they tried to visit her at home. However, she had gone missing, and seemed to be travelling around aimlessly by train. When she was finally examined by the psychiatric crisis service, she was paranoid, psychotic, and aggressive. A request to the mayor for emergency compulsory admission was granted.

## Discussion

### Summary of the findings

We found a common pattern of events preceding a compulsory admission. These include patients being psychotic and verbally or physically aggressive, stopping taking medication due to lack of insight, and avoiding contact with mental health-care professionals. These professionals might conceivably have been more alert and assertive with regard to the signs shown by these patients, and by patients’ tendency to avoid care. They could also have been more alert to patients’ family members.

### Limitations

A disadvantage of our approach to analysing the patient records is that it lacked the patients’ own perspective, which is not generally reported. It also omitted the perspective of caring family members. But the advantage of our approach is that it avoided selection bias – which, if we had chosen to interview patients, would probably have been present, as we would have been dependent on patients’ consent to participate in the study. Another limitation is that the health-care professionals’ perspective was studied in a specific context, i.e. the Dutch FACT context. Due to differences in cultural and practice norms, this perspective and how professionals report their observations and interpretations might be different in another context. We also focused primarily on finding the crucial factors preceding the compulsory admissions; the origins of these factors were not our scope. Establishing these origins may be the objective of another study. If so, it would require interviews with professionals and patients immediately after the admission.

As we analysed only compulsory admissions, leaving out voluntary admissions, we do not know whether the crucial factors identified in this study are unique to compulsory admissions, or whether some or all of them are also crucial to voluntary admissions. Yet, as we argued above under Background, we focused on compulsory admissions, because compulsory admissions have a strong effect on psychiatric patients and their relatives, because they are contrary to human rights and to the principles of shared decision-making and recovery-focused care, and because the overall numbers of compulsory admissions is rising. Some of these arguments might also be applicable to voluntary admissions, but clearly to a smaller extent.

One remarkable finding was that few patients were or on long-acting injectable antipsychotics or were assertively offered them. It could be argued that while a professional might not immediately detect a patient’s unreported cessation of oral medication, none would fail to detect a patient’s cessation of depot medication. In addition, there were few reports that other support agencies or primary care were involved. In principle, they could have played a role in supporting patients and protecting them against compulsory admission.

A final consideration is that a PRISMA factor was not included in the final synthesis unless it had been independently identified by both researchers in the individual PRISMA analyses. This meant that not all the PRISMA factors that were present were actually included in the final synthesis. However, because we were looking for crucial factors on the basis of a qualitative analytical method, our approach has the advantage of showing most relevant factors rather than all factors.

### Clinical implications

To our knowledge, this is the first study to provide qualitative data on the mental health-care process preceding compulsory admission. Unlike the factors regarding the health-care professionals, the factors regarding patient-related risk factors for compulsory admission were very much what one might expect. The question is thus why health-care professionals, who supposedly know well which risk factors are important for compulsory admissions, do not always act assertively enough on signs of relapse and care avoidance. Although the “A” in the abbreviation “FACT” stands for “assertive,” it seemed difficult for the teams that participated in this study actually to be assertive, at least with the patients included in this study. Professionals in the FACT teams have to divide their time between a smaller number of unstable patients who are difficult to engage (“true” ACT) [21]. and a larger number of relatively stable patients with severe mental illness who need lower-intensity, recovery-oriented care. In the former group of patients, professionals must be highly prepared to take an assertive position and to take over responsibility. In the latter group of patients, who are in the process of personal recovery and developing their own responsibility, this would be counter-therapeutic. The disadvantage of this flexible model may be that it is more difficult to be truly assertive when necessary. Additionally, most professionals in these FACT teams are working part-time, leading to differences in staffing during the week. Especially on Fridays, relatively few professionals share the work of the whole team. While the most urgent problems are taken care of, less urgent matters have to wait until the following week.

This may represent a more widespread problem: mental health-care professionals elsewhere may also find it difficult to apply assertive interventions creatively to patients with severe mental illness who show signs of relapse and care avoidance. But due to our focus on patients with a compulsory admission, we do not know whether the professionals were assertive towards other patients with similar characteristics, and may thus have prevented compulsory admissions.

It may be that psychiatric patients’ early signs of deterioration are not specific enough, and are masked too easily by the “noise” of daily practice. As a metaphor, these patient signals can be compared to yellow traffic lights at dangerous crossroads. Health-care workers’ sensitivity and patients’ sense of mastery may both be improved by the use of advance statements such as crisis plans, which focus on early signs and base preventive interventions upon them. This in turn might lead to timely recognition of these yellow traffic lights - and also to appropriate interventions [22].

## Conclusion

The health-care process preceding compulsory admission is complex, influenced as it is by acts, events, conditions and circumstances, failing barriers, and protective factors. Crucial patient factors in our study included psychosis, aggression, lack of insight, unauthorized reduction or cessation of medication, and care avoidance. Neither are health-care professionals as assertive as they could be in managing early signs of relapse and care avoidance.

## Additional file

Additional file 1: Anonymized example of a PRISMA tree of causes. (PDF 517 kb)

## Abbreviations

BOPZ: Exceptional admissions to psychiatric hospitals act (Dutch abbreviation)
FACT: Flexible assertive community treatment
MREC: Medical research ethics committee
PRISMA: Prevention and recovery system for monitoring and analysis
WGBO: Agreement on medical treatment act (Dutch abbreviation)
WMO: Medical research involving human subjects act (Dutch abbreviation)
