# Supplementary figures and images for: Crucial factors preceding compulsory psychiatric admission: a qualitative patient-record study
Source: BMC Psychiatry. 2017 Oct 24;17:350. doi: 10.1186/s12888-017-1512-y (PMC5655985; doi:10.1186/s12888-017-1512-y)

**Additional material:**

*Anonymized example of a*

*PRISMA tree of causes*

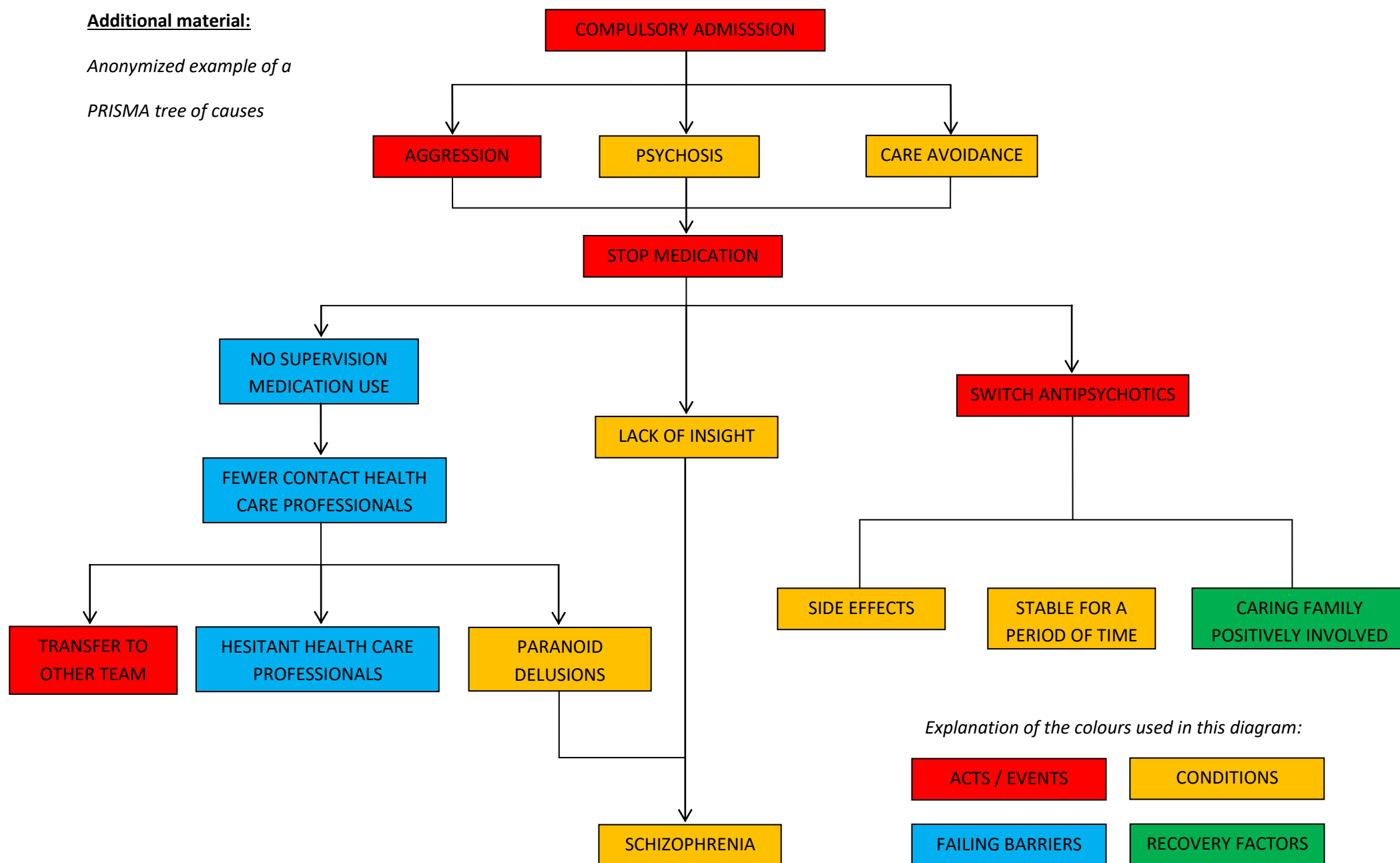

Supplement: Additional file 1: — Anonymized example of a PRISMA tree of causes. (PDF 517 kb) [file 12888_2017_1512_MOESM1_ESM.pdf]
